# Phytochemical Analysis and Profiling of Antitumor Compounds of Leaves and Stems of *Calystegia silvatica* (Kit.) Griseb.

**DOI:** 10.3390/molecules28020630

**Published:** 2023-01-07

**Authors:** Ahmed M. M. Youssef, Doaa A. M. Maaty, Yousef M. Al-Saraireh

**Affiliations:** 1 Department of Pharmacology, Faculty of Pharmacy, Mutah University, P.O. Box 7, Al-Karak 61710, Jordan; 2 Department of Botany and Microbiology, Faculty of Science, Al-Azhar University, Girls Branch, Cairo 11754, Egypt; doaamaaty1617.el@azhar.edu.eg; 3 Department of Pharmacology, Faculty of Medicine, Mutah University, P.O. Box 7, Al-Karak 61710, Jordan; yousef.sar@mutah.edu.jo

**Keywords:** cancers, cytotoxicity, *Calystegia Silvatica*, phytochemistry

## Abstract

Anti-tumor compounds from natural products are being investigated as possible alternatives for cancer chemotherapeutics that have serious adverse effects and tumor resistance. *Calystegia silvatica* was collected from the north coast of Egypt and extracted via methanol and *n*-hexane sub-fraction. The biologically active compounds of *Calystegia silvatica* were identified from the methanol and *n*-hexane extracts from the leaves and stems of the plant using GC-MS and HPLC. The antitumor properties of both parts of the plant were investigated against cancer and non-cancer cell lines using the MTT assay, and the IC_50_ in comparison to doxorubicin was calculated. The main compounds identified in the methanol extract were *cis*-vaccenic acid and *trans*-13-octadecenoic acid in the leaves and stems, respectively, and phenyl undecane and 3,7,11,15 tetramethyl-2-hexadeca-1-ol in the *n*-hexane extracts of the leaves and stems, respectively. Both parts of the plant contained fatty acids that have potential antitumor properties. The methanol extract from the stems of *C. silvatica* showed antitumor properties against HeLa, with an IC_50_ of 114 ± 5 μg/mL, PC3 with an IC_50_ of 137 ± 18 μg/mL and MCF7 with an IC_50_ of 172 ± 15 μg/mL, which were greater than Caco2, which had an IC_50_ of 353 ± 19 μg/mL, and HepG2, which had an IC_50_ of 236 ± 17 μg/mL. However, the leaf extract showed weak antitumor properties against all of the studied cancer cell lines (HeLa with an IC_50_ of 208 ± 13 μg/mL, PC3 with an IC_50_ of 336 ± 57 μg/mL, MCF7 with an IC_50_ of 324 ± 17 μg/mL, Caco2 with an IC_50_ of 682 ± 55 μg/mL and HepG2 with an IC_50_ of 593 ± 22 μg/mL). Neither part of the plant extract showed any cytotoxicity to the normal cells (WI38). Therefore, *C. silvatica* stems may potentially be used for the treatment of cervical, prostate and breast cancer.

## 1. Introduction

Cancer is one of the leading causes of mortality worldwide. Among surgery and radiation treatment modalities, chemotherapy remains the modality of choice for cancer therapy, particularly in patients with advanced stages of the disease [1,2,3]. However, the use of chemotherapeutic agents for cancer therapy is hindered because of severe long-term side effects and the development of drug resistance caused by inactivation and drug metabolism by different enzymes such as cytochromes P450 [4,5,6,7,8,9]. These factors limit the use of chemotherapeutic agents, making it a significant challenge in cancer therapy. Recent developments have enabled cancer researchers to better explore the potential use of natural products for cancer therapy. Fortunately, several natural products with various chemical structures and different pharmacological activities have shown promise in combating different cancers, these include alkaloids, terpenoids and flavonoids [6,10,11,12,13]. Therefore, collective efforts are needed to discover new therapeutic opportunities for cancer treatment arising from natural products.

The genus *Calystegia* R.Br., family *Convolvulaceae*, is represented by only one species—*Calystegia silvatica* subsp. *silvatica*—in Egypt. It is found in gardens and orchards in the Mediterranean region, Iran, western Europe and Australia [14]. It has been used for the treatment of fever, urinary tract disorders, constipation and reduced bile production (cholagogue) [15]. Additionally, it has been used for rheumatoid arthritis as an anti-inflammatory and pain killer [16,17], resolvent for pimples [18] and as an anti-tuberculosis treatment [16]. Lipids have reportedly been identified as the primary active components of the latex of *C. silvatica* [19]. Additionally, *C. sepium* is a species with strong relations to *C. silvatica,* which has been used to demonstrate the existence of resin glycosides known as calestegins [20,21]. The aerial parts of *C. sepium* have been used to isolate seven hexasaccharide resin glycosides, known as calsepins 1–7. Lung cancer cells (A549) showed cytotoxicity when exposed to calysepin 4 [22]. Two resin glycosides, calystegines A and B, were also isolated from the aerial parts of *Calystegia sepium*. Calystegine B showed an antitumor effect by preventing the assembly of tumor microtubule proteins of cancer cells [20,21]. Three cancer cell lines, including glioma cells (U87-MG), epidermal cell lines (A431) and breast cancer cells (MCF7), have been killed by a methanolic extract comprising the leaves of *C. sepium* compared to HGF-1 as normal cells [23]. There are no published data regarding the antitumor properties of *C. silvatica* in the literature; therefore, this is the first investigation of the antitumor effects of this plant against certain cancer cell lines.

## 2. Results

**Figure 1:**
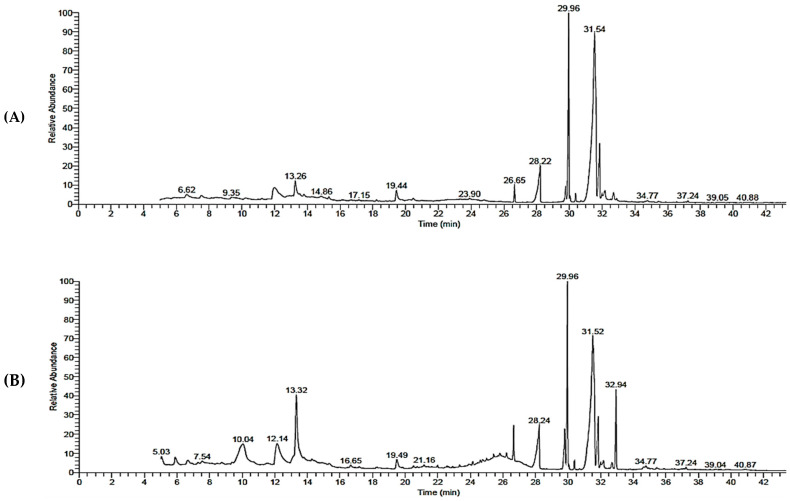
The spectra of methanol extract from *Calystegia silvatica* by gas chromatography–mass spectrometry: (**A**) leaves extract; (**B**) stems extract.

The compounds identified in the methanol extract from the leaves and stems of *C. silvatica* via gas chromatography–mass spectrometry (GC-MS) revealed fatty acids, phenols, sterols ketones and hydrocarbons. The fatty acids hexadecanoic acid, methyl ester (RA = 2.08% and RA = 3.60%), hexadecanoic acid (RA = 6.26% and RA = 6.30%), 9,12-Octadecadienoic acid, methyl ester (RA = 1.92% and RA = 4.63%), 9-octadecenoic acid, methyl ester (RA = 24.52% and RA = 18.64%), octadecanoic acid, methyl ester (RA = 1.05% and RA = 0.87%) and octadecanoic acid (RA = 10.71% and RA = 6.77%) were found in the leaves and stems, respectively (Table 1 and Figure 1). However, the fatty acids *cis*-5,8,11,14,17-eicosapentaenoic acid (RA = 0.8%) and *cis*-vaccenic acid (RA = 38.32%) were found in leaves, and trans-13-octadecenoic acid (RA = 25.06%) was found in stems. The iridoid glycoside proceroside (RA = 0.72%), 9-oxabicyclo [3.3.1]nonan-2-one, 5-hydroxy- (RA = 0.66%), essential oil 3,5-heptadienal, 2-ethylidene-6-methyl (RA = 2.82%), polyacetylene (S,Z)-Heptadeca-1,9-dien-4,6-diyn-3-ol (RA = 0.40%), organic aromatic compound naphthalenes 1,2-dihydro-1,5,8-trimethyl naphthalene (RA = 0.47%) and sesquiterpene alcohol cedran-diol, 8S,13- (RA = 0.35%) were found in the leaves. However, the ketone 2-nonanone, O-methyloxime (RA = 1.16%), 4H-pyran-4-one, 2,3-dihydro-3,5-dihydroxy-6-methyl (RA = 5.4%), benzofurans derivative 2,3-dihydro-benzofuran (RA = 5.53%), phenol 2-methoxy-4-vinylphenol (RA = 8.93%), aromatic compound 2,5-cyclohexadiene-1,4-dione (RA = 1.46%), nitrile derivative tetraacetyl-d-xylonic nitrile (RA = 0.59%), sterol 9,10-secocholesta-5,7,10 (19)-triene-3,24,25-triol (RA = 0.57%) and tributyl citrate (RA = 8.05%) were found in the stems (Table 1 and Figure 1).

**Figure 2:**
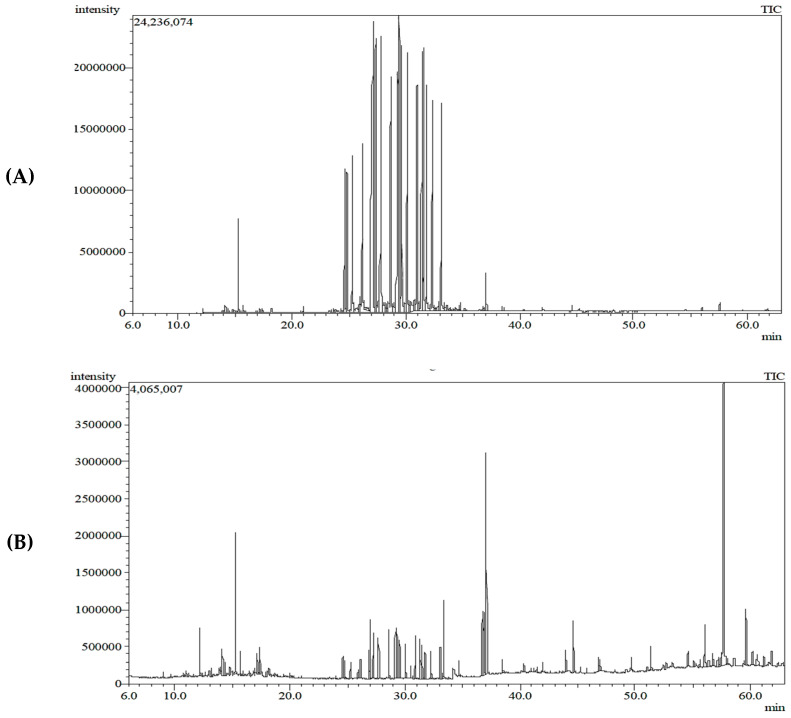
The spectra of *n*-hexane extract from *Calystegia silvatica* by gas chromatography–mass spectrometry: (**A**) leaves extract; (**B**) stems extract.

On the other hand, the major identified chemical constituents of lipids via the GC-MS analysis of an *n*-hexane extract from the leaves and stems of *C. silvatica* were alkane hydrocarbons undecane (with a relative abundance (RA) of 0.10% and 1.58%, respectively), methyl undecane (RA = 0.29% and RA = 1.85%, respectively), methyl dodecane (RA = 0.10% and RA = 1.99%, respectively), benzenes phenyl undecane (RA = 16.21% and RA = 4.65%, respectively), phenyl decane (RA = 10.11% and RA = 2.72%, respectively) and 2-phenyl tridecane (RA = 4.30% and RA = 1.15%, respectively) (Table 2 and Figure 2). The leaves and stems both contained fatty acids methyl ester 9,12, octadecanoic, methyl ester (RA = 0.10% and RA = 4.12%, respectively). The leaves and stems also contained the diterpene, 3,7,11,15 tetramethyl-2-hexadeca-1-ol (with a relative abundance of 0.53% and 8.10%, respectively), the phthalate ester bis (2-ethylhexyl) phthalate (RA = 0.1% and RA = 2.11%, respectively), beta-sitosterol acetate, a sterol, (RA = 0.10% and RA = 2.69%, respectively) and the sterol ergost-25-ene-3,5,6,12-tetrol (3.beta.,5.alpha.,6.beta.,12.beta.) (RA = 0.25% and RA = 0.76%, respectively). However, the octadecenoic acid, 12 hydroxy, methyl ester (RA = 0.44%), 9,12,octadecadienoic acid, 2 hydroxy-1 (hydroxy methyl) ethyl ester (RA = 0.91%) and hexadecenoic acid, 2-hydroxyl-1-(hydroxy methyl) ethyl ester (RA = 1.12%) were the fatty acids found in the stems, but they were not detected in the leaves. Campesterol (RA = 0.79%), stigmasterol (RA = 0.35%), obtusifiol (RA = 0.41%) and cholest-5-en-3-ol, 24-propylidene (RA = 0.40%) were the identified sterols found in stems only. Additionally, 9,19-cyclolanost-24-en-3-ol, (3 beta) (RA = 1.05%), betulinaldehyde (RA = 4.24%) and lanostan-3-ylacetate (RA = 1.1%) were the triterpenes found in the stems. Additionally, other compounds found in the stems were the oxygenated sesquiterpenes longifolenaldehyde (RA = 1.08%) and the diterpene thunbergol (RA = 0.85%). The only compound that was found in the leaves was 7-phenyl eicosane (RA = 0.99%), but it was not detected in the stems (Table 2 and Figure 2).

**Figure 3:**
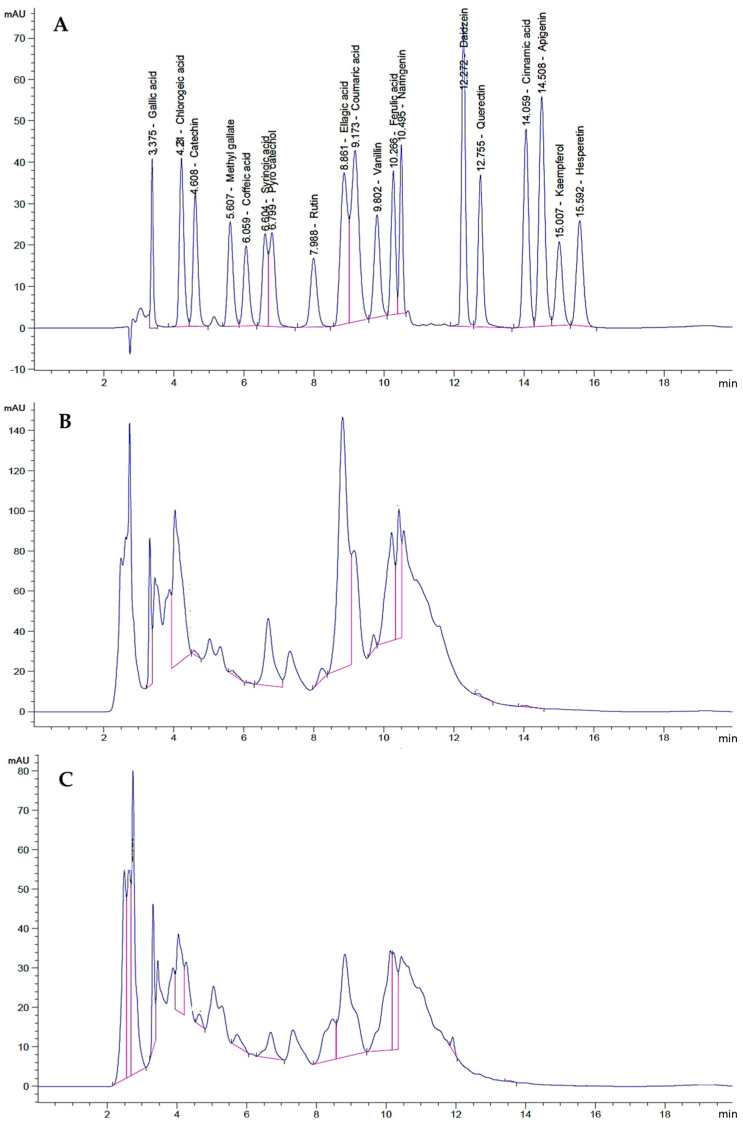
The HPLC chromatogram of methanol extract from *Calystegia silvatica*: (**A**) standard chromatogram; (**B**) leaves chromatogram; (**C**) stems chromatogram.

The total phenolic contents of the leaves and stems of *C. silvatica* were 21.18 mg and 17.30 mg GAE/g of dry weight (DW), respectively, and the total flavonoids were 7.10 mg and 12.45 mg CE/g DW, respectively. The HPLC was used to identify and quantify the phenolic compounds that exist in the methanolic extract from the leaves and stems of *C. silvatica.* (Table 3). As a result, phenolic acids, polyphenols, tannins, flavanones, flavonoids, isoflavone and carboxylic acids were identified in both parts of the plant. The leaves and stems contained the phenolic acid gallic acid, at a concentration of 1.05 mg/100 g dry weight (DW) and 0.5 mg/100 g DW, respectively, and the phenolic acid ferulic acid, at a concentration of 0.28 mg/100 g DW and 0.60 mg/100 g DW, respectively. The polyphenols found in the leaves and stems were catechin, at a concentration of 0.19 mg/100 g DW and 0.24 mg/100 g DW, respectively, and caffeic acid, at a concentration of 0.1 mg/100 g DW and 0.003 mg/100 g DW, respectively. Additionally, the phenolic compounds found in the leaves and stems were pyrocatechol, at a concentration of 3.14 mg/100 g DW and 0.36 mg/100 g DW, respectively, and coumaric acid, at a concentration of 2.40 mg/100 g DW and 0.19 mg/100 g DW, respectively. The phenylacrylate polyphenol compound chlorogenic acid was found in the leaves and stems at a concentration of 1.78 mg/100 g D.W and 0.42 mg/100 g DW, respectively. Additionally, the methyl gallate (gallate ester) was found in the leaves and stems at a concentration of 0.06 mg/100 g DW and 0.10 mg/100 g DW, respectively. The leaves and stems also contained tannin ellagic acid at a concentration of 0.69 mg/100 g DW and 0.21 mg/100 g DW, respectively. The benzaldehyde vanillin was found to have a concentration of 0.07 mg/100 g DW and 0.02 mg/100 g DW in the leaves and stems, respectively. The flavanone naringenin was found in the leaves and stems at a concentration of 0.67 mg/100 g DW and 0.08 mg/100 g DW, respectively. The monocarboxylic acid cinnamic acid was found to have the lowest concentration in the leaves and stems (0.01 mg/100 g DW and 0.001 mg/100 g DW, respectively), as compared to other identified phenolic compounds. However, the flavonoid quercetin was found in the leaves at a concentration of 0.05 mg/100 g DW, and the isoflavone daidzein was found in the stems at a concentration of 0.05 mg/100 g DW. Other phenolic compounds—syringic acid, apigenin, kaempferol and hesperetin—were not detected in the leaves or stems (Table 3 and Figure 3).

**Figure 4:**
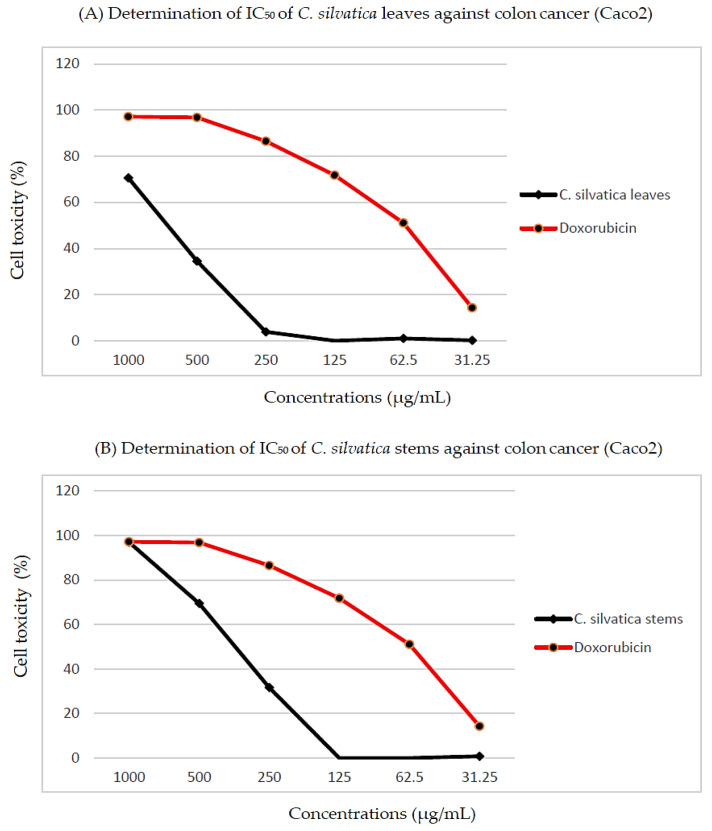
Determination of the half-maximal inhibitory concentration (IC_50_) of (**A**) *C. silvatica* leaves extract and (**B**) *C. silvatica* stems extract against colon cancer (Caco2).

The percentage of cytotoxicity was represented as [cell viability percentage – 100] and plotted on the *Y*-axis; the concentration of the extracts from leaves and stems was plotted on the *X*-axis to calculate the half-maximal inhibitory concentration (IC_50_). For example, the IC_50_ of the leaves extract against colon cancer was 682 ± 55 μg/mL, and it was 353 ± 19 μg/mL for the stems extract (Figure 4).

The antitumor properties of *C. silvatica* leaves and stems against colon cancer (Caco2), cervical cancer (HeLa), prostate cancer (PC3), breast cancer (MCF7), hepatocellular carcinoma (HepG2) and normal human fetal lung fibroblast (WI38) cell lines were studied using an MTT assay. Dunnett’s test was used to compare the calculated IC_50_ values of the leaves and stems extracts to the positive control, doxorubicin. Based on the NCI criteria, the methanol extract from the leaves and stems exhibited different cytotoxicities against all of the cancer cell lines (Table 4). The antitumor properties of *C. silvatica* stems against cervical cancer (HeLa) (114 ± 5 μg/mL), prostate cancer (PC3) (137 ± 18 μg/mL) and breast cancer (MCF7) (172 ± 17 μg/mL) were moderate compared to doxorubicin. However, the antitumor properties of stems against colon cancer (CaCo2) (353 ± 19 μg/mL) and hepatocellular carcinoma (HepG2) (236 ± 17 μg/mL) were weak compared to the positive control. In contrast, the cytotoxicities of leaves on cervical cancer (HeLa) (IC_50_ = 208 ± 13 μg/mL), prostate cancer (PC3) (IC_50_ = 336 ± 57 μg/mL) and breast cancer (MCF7) (IC_50_ = 324 ± 17 μg/mL) were weak compared to doxorubicin. However, the leaves did not show any antitumor properties against colon cancer (Caco2) (IC_50_ = 682 ± 55 μg/mL) or hepatocellular carcinoma (IC_50_ = 593 ± 33 μg/mL). Interestingly, neither the extract from leaves nor from stems showed any cytotoxicity to normal human fetal lung fibroblast (WI38) with an IC_50_ of 543 ± 33 μg/mL and 408 ± 4 μg/mL, respectively, compared to the positive control. The selectivity index of the methanol extract from *C. silvatica* leaves and stems was estimated as described above. Therefore, no cytotoxic selectivity was shown for the leaves extract from *C. silvatica* in any of the studied cancer cell lines (values < 3). However, the stems extract showed cytotoxic selectivity in HeLa (3.5) and prostate cancer (3) but not in colon cancer (1.7), breast cancer (1.1) or hepatocellular carcinoma (2.3) (Table 5).

**Figure 5:**
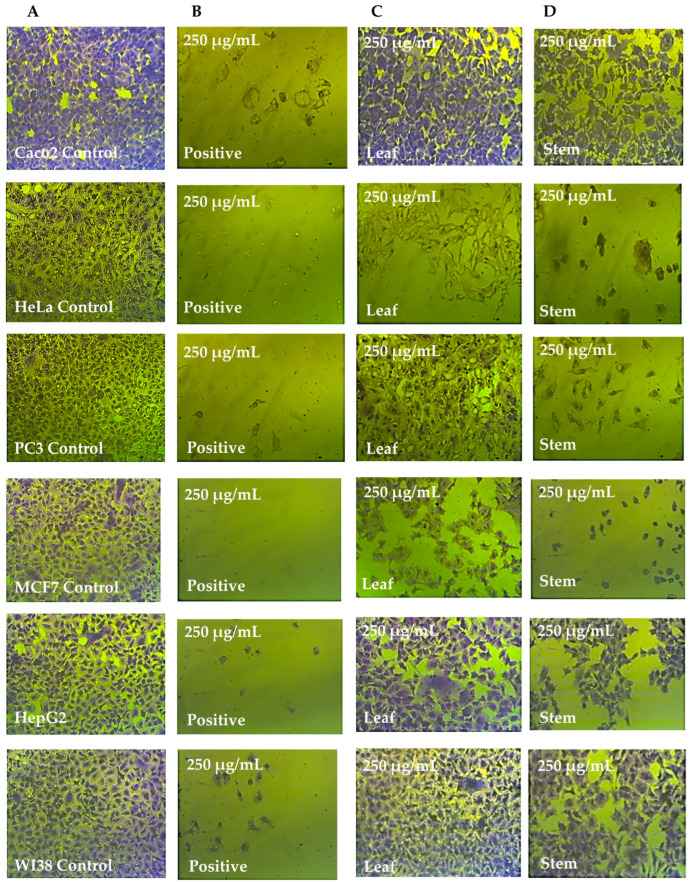
Anticancer effects of *Calystegia silvatica* leaves and stems extracts in methanol on cancer cell lines: (**A**) complete monolayer sheets are seen in all of the cancer cell lines that have not been treated; (**B**) doxorubicin treatment results in rounded and shrunk cells in all of the cancer cell lines; (**C**) shrunk cells are observed in HeLa and MCF7 cell lines treated with the extract from *Calystegia silvatica* leaves; however, no morphological changes are observed in Caco2, HepG2 and WI38; (**D**) the extract from *Calystegia silvatica* stems used to treat HeLa, PC3, MCF7 and HepG2 cancer cell lines revealed significantly rounded and shrunk cells; however, small rounded and shrunk cells are observed in Caco2 and W138.

**Figure 6:**
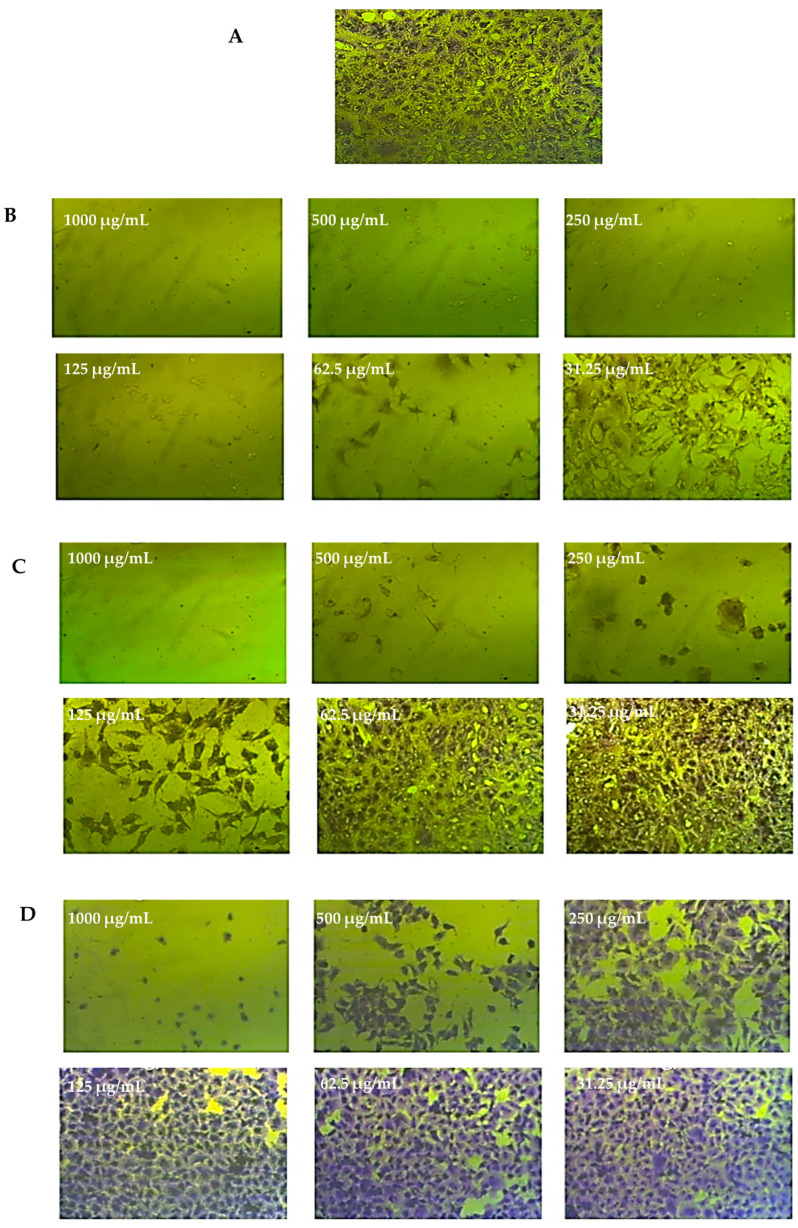
An example of the moderate anticancer effect of the stems extract from *Calystegia sylvatica* against cervical cancer cell lines (HeLa): (**A**) complete monolayer sheets of cervical cancer cells (HeLa) that have not been treated; (**B**) the effect of doxorubicin treatment at different concentrations; (**C**) the effect of the stems extract from *C. sylvatica* against HeLa cell lines at different concentrations; (**D**) the effect of the stems extract from *C. sylvatica* against normal human fetal lung fibroblast (WI38).

**Figure 7:**
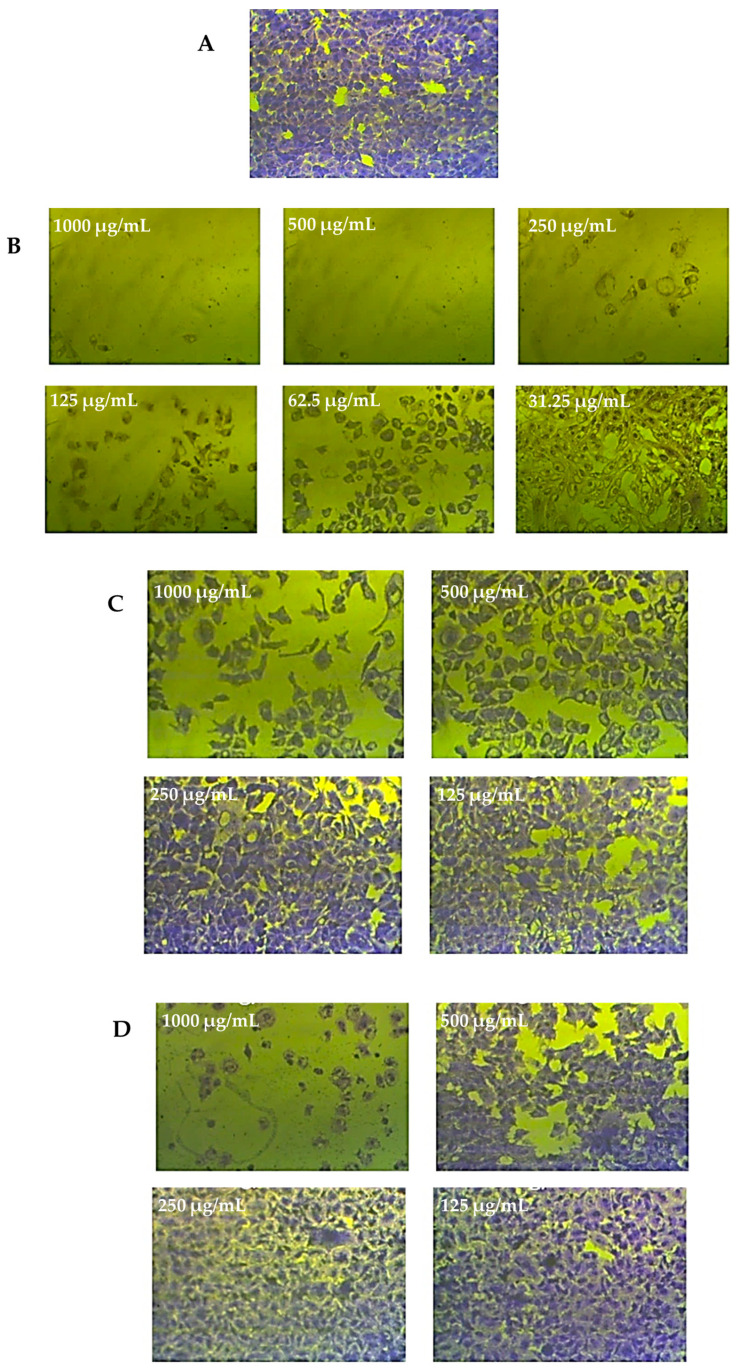
An example of the weak anticancer effect of the leaves extract from *Calystegia sylvatica* against colon cancer cell lines (Caco2): (**A**) complete monolayer sheets of colon cancer cell lines (Caco2) that have not been treated; (**B**) the effect of doxorubicin treatment at different concentrations; (**C**) the effect of the leaves extract from *C. sylvatica* against Caco2 cell lines at different concentrations; (**D**) the effect of the leaves extract from *C. sylvatica* against normal human fetal lung fibroblast (WI38) at different concentrations.

The different cell lines were treated for 72 h with 250 μg/mL of extract from *C. silvatica* leaves and stems and were microscopically examined. The methanol extract from the leaves of *C. silvatica* caused a small shrinkage to HeLa and MCF7 cell lines only, while the stems extract caused a significant shrinkage to HeLa, PC3 and MCF7 cancer cell lines, which became rounded and detached. However, Caco2 and HepG2 did not show any change in morphology compared to untreated control cells, and WI38 cell lines did not exhibit any change in morphology when treated with either the leaves or stems of *C. silvatica* compared to their control cells (Figure 5, Figure 6 and Figure 7).

## 3. Discussion

The GC-MS results of the methanol extract from the leaves and stems of *C. silvatica* revealed the existence of potential antitumor composites. For example, the 4H-pyran-4-one, 2,3-dihydro-3,5- dihydroxy-6-methyl found in the stems has been reported to possess an antiproliferative effect [24]. The stems also contained benzofuran derivatives 2,3-dihydro-benzofuran, which has also been reported to have antitumor properties [25]. Additionally, the tetra acetyl-d-xylonic nitrile detected in stems was found to have antitumor and antioxidant properties [26]. The two fatty acids, hexadecanoic acid, methyl ester and hexadecanoic acid, have not been evaluated for their antitumor properties; however, they have been reported to display antioxidant activity [27,28]. However, the fatty acids 9,12-octadecadienoic acid, methyl ester and 9-octadecenoic acid, methyl esters, have been reported to have antitumor and antioxidant properties [29,30], and octadecanoic acid, methyl ester and octadecanoic acid and *cis*-5,8,11,14,17-eicosapentaenoic acid have been reported to have antitumor properties [31,32]. Interestingly, these compounds were found in significant amounts in the methanol extract from the leaves and stems of *C. silvatica,* except *cis*-5,8,11,14,17-eicosapentaenoic acid, which was only detected in the leaves. c*is*-Vaccenic acid and *trans*-13-octadecenoic acid were also reported to have antitumor properties [33,34]. Importantly, *cis*-vaccenic (RA = 38.32%) and *trans*-13-octadecenoic acid (RA = 25.06%) (Table 1 and Figure 1) were found in the leaves and stems, respectively, in a greater amount than other detected compounds of methanol extract from *C. silvatica* (Table 1 and Figure 1). The polyacetylene (S,Z)-heptadeca-1,9-dien-4,6-diyn-3-ol has been reported to have antitumor properties [35]. The phenol compound, 2-Methoxy-4-vinylphenol, has not been evaluated for antitumor properties; however, it has been reported to have anti-inflammatory properties [36]. Additionally, the organic compound (vitamin D with 3 hydroxyl (OH) groups) 9,10-secocholesta-5,7,10(19)-triene-3,24,25-triol has not been evaluated for its antitumor properties, though it has been reported to have antibacterial properties [37]. Moreover, the sesquiterpene alcohol cedran-diol, 8S,13- has not been reported to have antitumor properties; however, it has been reported to have antimicrobial and anti-inflammatory properties [38]. The ketone 2-nonanone, O-methyloxime has not been evaluated for biological activities until now. The 2,5-cyclohexadiene-1,4-dione and tributyl citrate have also not been evaluated for biological activities. Additionally, the steroid proceroside, essential oil 3,5-Heptadienal, 2-ethylidene-6-methyl, the naphthalene derivative 1,2-dihydro-1,5,8-trimethyl naphthalene and 9-oxabicyclo[3.3.1]nonan-2-one, 5-hydroxy- have not been evaluated for biological activities until now.

On the other hand, the identified compounds of the *n*-hexane extract from the leaves and stems of *C. silvatica* were reported to have antitumor properties. The diterpene 3,7,11,15-tetramethyl-2- hexadeca-1-ol (RA = 8.10%) has been reported to have antitumor properties [39], which has a higher concentration in the stems than leaves and among other compounds identified in stems. Additionally, the phthalate ester bis (2-ethylhexyl) phthalate (RA = 2.11%) has been reported to have antitumor and antioxidant properties [40], and it has a higher concentration in the stems than in leaves. Additionally, campesterol [41], stigmasterol [42], beta-sitosterol acetate [43,44] and obtusifoliol [45] are sterols that are found in the stem and have been reported to have antitumor properties. Betulinaldehyd is a triterpene also found in the stems and has been reported to possess antitumor properties [46]. Importantly, these detected antitumor compounds were found only in the stems of an *n*-hexane fraction of *C. silvatica* (Table 2). Therefore, this may explain the greater antitumor effect of stems against cervical cancer (IC_50_ = 114 ± 5 μg/mL), prostate cancer (IC_50_ = 137 ± 18 μg/mL) and breast cancer (IC_50_ = 172 ± 15 μg/mL) compared to leaves that showed an antitumor effect against cervical cancer (IC_50_ = 208 ± 13 μg/mL), prostate cancer (IC_50_ = 336 ± 57 μg/mL) and breast cancer (IC_50_ = 324 ± 17 μg/mL) (Table 3 and Figure 4). Additionally, the stems extract showed SI values of 3 and 3.5 against HeLa and PC3, respectively, this may indicate that the antitumor properties of stems against cancerous cells were greater than non-cancerous (WI38) cells (Table 4). However, thunbergol is an identified diterpene that has not been evaluated for antitumor properties, but it has been reported to have antimicrobial properties [47]. Additionally, the ergost-25-ene-3,5,6,12-tetrol (3.beta.,5.alpha.,6.beta.,12.beta.) and cholest-5-en-3-ol, 24-propylidene, (3 beta) are identified sterols that have not been evaluated for antitumor properties; however, they have been reported to have antioxidant properties [48] and antimicrobial properties [49], respectively. Additionally, the triterpene 9,19-Cyclolanost-24-en-3-ol, (3.beta.) has not been evaluated for antitumor properties; however, it has been reported to have antioxidant and antimicrobial properties [50] (Table 2 and Figure 2). The 9,12,octadecadienoic acid, 2 hydroxy-1 (hydroxy methyl) ethyl ester and hexadecenoic acid, 2-hydroxyl-1-(hydroxy methyl) ethyl ester are identified fatty acids of *n*-hexane extract that have not been evaluated for antitumor properties; however, they have been reported to have antioxidant properties [51,52]. The nonadec-1-ene is an essential oil that has not been evaluated for antitumor properties; however, it has been reported to display antimicrobial and antioxidant activity [53]. The alkane hydrocarbons methyl undecane, methyl dodecane and the benzenes 2-phenyl decane, 6-phenyl decane, 7-phenyl eicosane, phenyl undecane and 2-phenyl tridecane have not been evaluated for biological activities; however, methyl undecane has been reported to have anti-inflammatory properties [54]. Additionally, some compounds identified in the *n*-hexane extract that have not been evaluated for biological activities until now are the fatty acid octadecenoic acid, 12 hydroxy, methyl ester, the triterpenoid lanostan-3-ylacetate and the oxygenated sesquiterpenes longifolenaldehyde.

The HPLC analysis for phenolic compounds in the methanol extract revealed that the leaves and stems extracts contained several phenolic and flavonoid compounds that could potentially contribute to the antitumor effect of *C. silvatica* against the studied cancer cell lines. Gallic acid is an identified phenolic acid that has been reported to have an antitumor effect [55]. Additionally, chlorogenic acid is a phenylacrylate polyphenol compound that has been reported to have antitumor properties [56]. The identified polyphenols with antitumor properties in *C. silvatica* extract are catechin [57] and caffeic acid [58]. Methyl gallate (gallate ester) has also been reported to have antitumor properties [59]. The identified phenolic compounds with antitumor properties are coumaric acid [60], ferulic acid [61] and pyrocatechol [62]. The vanillin is an identified benzaldehyde that has been reported to possess antitumor properties [63]. Additionally, ellagic acid is a tannin and has been reported to have antitumor properties [64]. Naringenin is a flavanone that has been reported to have antitumor properties [65]. Additionally, the flavonoid quercetin found in the leaves has been reported to have antitumor properties [38], and the isoflavone daidzein found in the stems has also been reported to have antitumor properties [66]. The identified monocarboxylic acid cinnamic acid has been reported to have antitumor properties [67].

*C. sepium*, which has a close affiliation to *C. silvatica*, showed an antitumor effect against breast cancer cells (MCF7), epidermal cell line (A431) and glioma cell line (U87-MG) compared to HGF-1 as normal cells [23]. These findings are in line with our MTT assay results in which the methanol extract from *C. silvatica* stems demonstrated an antitumor effect against breast cancer cell lines (MCF7) without cytotoxicity to the normal human fetal lung fibroblast (WI38). This study showed that stem extracts of *C. silvatica* have a higher antitumor effect than leaf extracts against most of the tested cancer cells without affecting the non-cancer cells. This may be due to the number of compounds that have antitumor properties found in the stems more than in the leaves. Therefore, the extract from the stems of *C. silvatica* may have potential antitumor properties against cervical, prostate and breast cancer but not against colon cancer and hepatocellular carcinoma; however, the extract from the leaves has weak antitumor effects. 

## 4. Materials and Methods

### 4.1. Plant Material 

Aerial parts of *C. silvatica* in the flowering stage were collected from the Al Alamein region, which is about 101 km from Alexandria, Egypt. Dr. Iman Al-Gohary from the Desert Research Centre (DRC), Cairo, Egypt, identified the samples. Voucher specimens were placed in the Center’s Herbarium (CAIH-0042R). Both parts of the plant were washed using tap water and dried for 10 days at 25 °C in a ventilated room in the shade and were subsequently powdered separately [68]. 

### 4.2. Extraction and Fractionation

#### 4.2.1. Methanol Extract Preparation

Two hundred grams of the air-dried powdered plant leaves and stems was extracted using the cold percolation technique. The extract was then placed on an orbital shaker for 72 h at 25 °C using 500 mL of 70% methanol three times (500 mL each time). A Buchner funnel was used to filtrate the methanol extracts. Then, the methanol was completely eliminated from the methanol extracts via concentration under reduced pressure at 40 °C and a rotary evaporator. In a dissector, the residues were dried to provide dry weights of 24.68 g/100 g for the leaves and 14.93 g/100 g for the stems. The bioactive components were identified using gas chromatography-mass spectrometry (GC-MS) analysis of the crude methanol extracts [68,69].

#### 4.2.2. GC-MS Analysis for the Methanol Extract

A Thermo Scientific TRACE 1310 gas chromatograph was coupled to an ISQLT single quadrupole mass spectrometer. The ionization voltage was 70 eV, the ionization mode was EI and the column was DB5-MS, 30 m, 0.25 mm ID (J & W Scientific, Folsom, CA, USA). The temperature program was as follows: 40 °C for 3 min, 280 °C for 5 min, 5 °C/min to 290 °C (held for 1 min) and then static at 7.5 °C/min. The injector temperature was 200 °C, the detector was 300 °C and the flow rate of the carrier gas, helium, was 1 mL/min. The WILEY and NIST Mass Spectral Databases were employed as search libraries [68]. 

#### 4.2.3. *n*-Hexane Sub-Fraction Extract Preparation 

The lyophilized crudes of the methanol extract from 2.5 g of leaves and stems were redissolved in 250 mL of distilled water. Then, a separatory funnel was used to partition the redissolved crude with *n*-hexane for 24 h. Utilizing a rotary evaporator at 40 °C, the *n*-hexane sub-fraction was condensed to dryness at reduced pressures. The dried fractions of leaves and stems were 1.3 g and 0.86 g, respectively, and they were analyzed using GC-MS [11,69].

#### 4.2.4. GC-MS Analysis for *n*-Hexane Sub-Fraction Extract

The Shimadzu GCMS-QP2010 (Tokyo, Japan) is equipped with a split/splitless injector and Rtx-5MS fused bonded column (30 m, 0.25 mm i.d., 0.25 m film thickness) from Restek (Bellefonte, PA, USA), which were utilized to record the mass spectra. The starting column temperature was raised to 300 °C at a rate of 5 °C/min for 5 min and was then maintained there for 2 min (isothermal). The temperature of the injector was 250 °C. The helium carrier gas flowed at a rate of 1.41 mL/min. All mass spectra were recorded using the following settings: 60 mA filament emission current, 70 eV ionization voltage and a 200 °C ion source, and the split mode injections of samples that were diluted (1% *v/v*) were carried out (split ratio 1:15) [69].

#### 4.2.5. Total Phenolic and Flavonoid Determination

The Folin–Ciocalteu method was used to quantify the phenolic compounds [11,70]. A volume of 0.25 mL of the Folin–Ciocalteu reagent (2 N) was added to 0.2 mL of the 80% methanolic extract in a volumetric flask (10 mL). Saturated sodium carbonate (1 mL) (20% in distilled water) was added after three minutes, and the volume was completed with distilled water. A Unicam UV-visible Spectrometer was used to measure the absorbance of blue color after 1 h at λ_max_ 725 nm against a blank (distilled water). Gallic acid was used to obtain a standard calibration curve. The results were represented as milligrams of gallic acid equivalents (GAE) per gram of dry weight (D.W) [11,70].

An aluminum chloride colorimetric method was used to quantify the total flavonoid content [11,70]. Distilled water with a volume of 1.25 mL was used to dilute the methanolic extract (0.25 mL). A 5% NaNO_2_ solution was then added to the mixture in a volume of 75 µL. After 6 min, 150 µL of a 10% AlCl_3_.6H_2_O solution was added, and the combination was left to stand for an additional 5 min. Then, 0.5 mL of a 1 M NaOH solution was added, and the mixture was then completed with 2.5 mL of distilled water. The absorbance was measured at λ_max_ 510 nm against the blank (distilled water). The (+)-catechin was used to obtain a standard calibration curve. The results were represented as milligrams of catechin equivalents (CE) per gram of dry weight.

#### 4.2.6. HPLC Analysis of Phenolic Compounds

##### Standards

HPLC reagents: methanol and acetonitrile (HPLC grade) were purchased from SDS (Peypin, France), and trifluoroacetic acid was purchased from Merck (Darmstadt, Germany) for phenolic compound analysis. Milli- Q (Millipore, MA, USA) was used to provide distilled water. Phenolic standards: gallic acid, chlorogenic acid, catechin, methyl gallate, caffeic acid, syringic acid, pyrocatechol, rutin, ellagic acid, coumaric acid, vanillin, ferulic acid, naringenin, daidzein, quercetin, cinnamic acid, apigenin, kaempferol and hesperetin were obtained from Sigma Co. (St. Louis, MO, USA). All phenolic standards had a 98% purity level.

##### Quantitative analysis of phenolic compounds via HPLC

The previously prepared methanolic extract from the leaves and stems of *C. silvatica*. (200 mg) was dissolved in HPLC-grade acetonitrile (2 mL). An Agilent 1260 series reverse-phase HPLC apparatus (Agilent, USA) was used for phenolic compound analysis. The separation of phenolic compounds was performed using an Eclipse C18 column (4.6 mm × 250 mm i.d., 5 μm). The mobile phase consisted of water (A) and 0.05% trifluoroacetic acid in acetonitrile (B) at a flow rate of 0.9 mL/min. The mobile phase was programmed consecutively in a linear gradient as follows: 0 min (82% A); 0–5 min (80% A); 5–8 min (60% A); 8–12 min (60% A); 12–15 min (82% A); 15–16 min (82% A); 16–20 min (82%A). The samples were monitored at 280 nm using a multi-wavelength detector. The injection volume was 5 μL for each of the sample solutions. The column temperature was maintained at 40 °C. Standard flavonoids and phenolic acids were prepared as 10 mg/50 mL stock solution in methanol. They were then diluted to reach working concentrations (20–40 µg/mL) and injected into HPLC. The quantities of the detected compounds were expressed as µg/g, and the peak area of the external standards was employed to quantify the phenolic compounds in the methanol extracts from the leaves and stems [11,70].

### 4.3. Antitumor Properties Evaluation 

The cell lines laboratory at Vacsera, Dokkey, Giza, Egypt provided colon cancer (Caco2), cervical cancer (HeLa), prostate cancer (PC3), breast cancer (MCF7), hepatocellular carcinoma (HepG2) and normal human fetal lung fibroblast (WI38). 

#### 4.3.1. Culturing

The sterility of the process was maintained using a laminar airflow cabinet. The Roswell Park Memorial Institute medium (RPMI 1640) was used to sustain the cell culture. A mixture of one percent of antibiotic and antimycotic (10,000 μg/mL streptomycin sulphate, 25 μg/mL amphotericin B and 10,000 U/mL potassium penicillin) and 1% L-glutamine were added to the medium. The 10% heat-inactivated fetal bovine serum was used to supplement the medium [70].

#### 4.3.2. MTT Assay

The 3-(4,5-dimethylthiazol-2-yl)-2,5-diphenyl-2H-tetrazolium bromide (MTT) assay was employed to measure cytotoxicity. A purple formazan was created from the yellow MTT via a mitochondrial reduction [70]. For inoculation, a 96-well microplate was filled with 1 × 10^5^ cells per well in 100 µL of Roswell Park Memorial Institute medium (RPMI 1640). Conditions of 5% CO_2_, 37 °C and 95% humidity were used to incubate the microplate for 24 h to produce a completely formed monolayer sheet. After the cells formed a confluent layer, the growth media was decanted from 96-well microplates. Dimethyl sulfoxide (0.1%) was used to dissolve the methanol extract from the leaves and stems. The dissolved extract was serially diluted using a growth medium to achieve final concentrations of are: 31.25, 62.5, 125, 250, 500 and 1000 µg/mL [70]. Confluent cell monolayers were injected with 0.1 mL of the extract at each concentration using a multichannel pipette, and then they were dispersed throughout the 96 wells. The cells that were treated with the extracts were incubated at 37 °C and 5% CO_2_ for 24 h. For each extract concentration, three wells were used. Control cells were incubated without the extracts from the leaves and stems. Phosphate-buffered saline (Bio Basic Canada Inc., Markham, ON, Canada) was used to dissolve the MTT powder to provide a solution with a 5 mg/mL concentration. Each well received 20 µL of the MTT solution following the completion of the incubation period. A shaker (MPS-1, Biosan, London, UK) was used for mixing, which was performed at 150 rpm for 5 min. The 96-well microplate was then kept for 4 h at 37 °C with 5% CO_2_. A metabolic byproduct of the MTT called formazan was resuspended in 200 µL of DMSO and aggressively shaken for five minutes at 150 rpm. The optical density at 560 nm was determined using a microplate reader. A background reference wavelength of 620 nm was used to adjust the results. All experiments were performed in triplicate.

#### 4.3.3. Determination of IC_50_ Values 

Using GraphPad Prism version 7 (GraphPad software Inc., San Diego, CA, USA), the values of the varying concentrations of the IC_50_ of the methanol extract from *C. silvatica* and doxorubicin (as a positive control) against colon cancer (Caco2), cervical cancer (HeLa), prostate cancer (PC3), breast cancer (MCF7), hepatocellular carcinoma (HepG2) and normal human fetal lung fibroblast (WI38) cell lines were computed. Equation (1) was used to determine the percentage of growth inhibition as follows [70]:

Growth Inhibition (%) = 100 − (mean OD of individual test group/mean OD of control group) × 100(1)

#### 4.3.4. Criteria for Antitumor Effect Levels 

The level of cytotoxicity of the methanol extract from *C. silvatica* was categorized using the Geran protocol and the guidelines of the National Cancer Institute (NCI) of the USA as follows: highly cytotoxic substances (IC_50_ ≤ 20 µg/mL), moderately cytotoxic substances (IC_50_ of 21–200 µg/mL), weakly cytotoxic substances (IC_50_ of 201–500 µg/mL) and non-cytotoxic substances (IC_50_ > 501 µg/mL) [71,72].

#### 4.3.5. Selectivity Index

The ratio of a plant extract’s IC_50_ value in a non-cancer cell line (WI38) to its IC_50_ value in each cancer cell line is known as the selectivity index. SI values below 3 indicate that the extract is not selective to non-cancer cells [71]. The selectivity indices of the methanol extract were determined using the following Equation (2):

Selectivity Index = (IC_50_ of non-cancer cell line (WI38))/(IC_50_ of a cancer cell line) (2)

#### 4.3.6. Microscope 

The morphological structures of cell lines were examined using a Nikon 11,881 inverted microscope with objective 8× at various methanol *C. silvatica* extract concentrations.

## 5. Conclusions

The methanolic and *n*-hexane extracts from the leaves and stems of *C. silvatica* contained a diverse variety of phytochemicals that were identified by GC/MS and HPLC. Most of these phytochemicals had antitumor properties. Most of the phytochemicals that possessed antitumor properties were greater in the stems than in the leaves. Therefore, the antitumor effect of the stems of *C. silvatica* on cervical cancer (HeLa), prostate cancer (PC3), breast cancer, colon cancer (Caco2) and hepatocellular carcinoma (HepG2) was greater than that of the leaves, which showed a weak antitumor effect against all of the studied cancer cell lines. The normal cells were not affected by the cytotoxic activities of either part of the *C. silvatica* plant. Therefore, the isolation of the bioactive compounds from the stems of *C. silvatica* and an investigation of their antitumor properties against different cancer cell lines may be required in future work. Additionally, the antitumor properties and a phytochemical investigation of *C. silvatica* have never been reported before. This study will thus serve as the basis for this plant, and more pharmacological investigations are recommended.

## Figures and Tables

**Table 1:** Biological compound identification of methanol extract from *Calystegia silvatica* leaves and stems using GC/MS.

No.					Leaves		Stems	
	Compounds	MW	M.F.	Category	Rt	RA%	Rt	RA%
1	2-nonanone, O-methyloxime	171	C_10_H_21_NO	Ketone			5.88	1.16
2	4H-pyran-4-one, 2,3-dihydro-3,5-dihydroxy-6-methyl	144	C_6_H_8_O_4_	Aromatic organic compound			10.04	5.4
3	2,3-dihydro-benzofuran	120	C_8_H_8_O	Benzofurans derivatives			12.12	5.53
4	2-Methoxy-4-vinylphenol	150	C_9_H_10_O_2_	Phenol aromatic organic compound			13.32	8.93
5	2,5-cyclohexadiene-1,4-dione	180	C_10_H_12_O_3_	Aromatic organic compound			19.49	1.46
6	Tetraacetyl-d-xylonic nitrile	343	C_14_H_17_NO_9_	Aromatic nitro compound			26.22	0.59
7	Hexadecanoic acid, Methyl ester	270	C_17_H_34_O_2_	Fatty acid methyl ester	26.65	2.08	26.65	3.60
8	Hexadecanoic acid	256	C_16_H_32_O_2_	Fatty acid	28.21	6.26	28.23	6.30
9	9,12-Octadecadienoic acid, methyl Ester	294	C_19_H_34_O_2_	Fatty acid methyl ester	29.78	1.92	29.80	4.63
10	9-Octadecenoic acid, methyl ester	296	C_19_H_36_O_2_	Fatty acid methyl ester	29.95	24.53	29.96	18.64
11	Octadecanoic acid, Methyl ester	298	C_19_H_38_O_2_	Fatty acid methyl ester	30.39	1.05	30.39	0.87
12	*trans*-13-Octadecenoic acid	282	C_18_H_34_O_2_	Fatty acid			31.52	25.06
13	Octadecanoic acid	284	C_18_H_36_O_2_	Fatty acid	31.85	10.71	31.85	6.77
14	9,10-secocholesta-5,7,10(19) -triene-3,24,25-triol	416	C_27_H_44_O_3_	Sterol			32.00	0.57
15	Tributyl citrate	360	C_18_H_32_O_7_	Carbonyl compound			32.49	8.05
16	Proceroside	548	C_29_H_40_O_10_	Iridoid glycoside	6.62	0.72		
17	9-Oxabicyclo [3.3.1]nonan-2-one, 5-hydroxy	156	C_8_H_12_O_3_	Bicyclo aromatic compound	7.52	0.66		
18	3,5-Heptadienal, 2-ethylidene-6-methyl	150	C_10_H_14_O	Essential oil	13.26	2.82		
19	(S,Z)-Heptadeca-1,9-dien-4,6-diyn-3-ol	244	C_17_H_24_O	Polyacetylene	13.79	0.40		
20	1,2-dihydro-1,5,8-trimethyl Naphthalene	172	C_13_H_16_	Naphthalenes derivatives	15.31	0.47		
21	Cedran-diol, 8S,13	238	C_15_H_26_O_2_	Sesquiterpene	20.46	0.35		
22	*cis*-Vaccenic acid	282	C_18_H_34_O_2_	Omega-7 fatty acid	31.55	38.32		
23	*cis*-5,8,11,14,17-Eicosapentaenoic Acid	302	C_20_H_30_O_2_	Fatty acid	31.99	0.80		

**Table 2:** Biological compound identification of *n*-hexane extract from *Calystegia silvatica* leaves and stems using GC/MS.

No.					Leaves		Stems	
	Compounds	MW	M.F.	Category	Rt	RA%	Rt	RA%
1	Undecane	156	C_11_H_24_	Alkane hydrocarbon	12.16	0.10	12.15	1.58
2	Methyl undecane	170	C_12_H_26_	Branched alkane hydrocarbon	14.02	0.29	14.15	1.85
3	Methyl dodecane	184	C_13_H_28_	Branched alkane hydrocarbon	17.16	0.10	17.16	1.99
4	2-Phenyl decane	218	C_16_H_26_	Benzene	23.50	10.11	24.60	2.72
5	6-phenyl decane	232	C_18_H_3_O	Benzene	25.73	0.28	20.17	1.71
6	7-phenyl eicosane	358	C_26_H_46_	Eicosyl benzene	25.93	0.99		
7	Phenyl undecane	232	C_17_H_28_	Benzene	26.78	16.21	26.91	4.65
8	2-phenyl tridecane	260	C_19_H_32_	Benzene	33.15	4.30	33.07	1.15
9	Octadecenoic acid, 12 hydroxy, methyl ester	312	C_19_H_36_O_3_	Fatty acid methyl ester			40.34	0.44
10	9,12,octadecadienoic acid, 2 hydroxy-1(hydroxy methyl) ethyl ester	354	C_21_H_38_O_4_	Fatty acid ethyl ester			46.81	0.91
11	Hexadecenoic acid, methyl ester	270	C_17_H_34_O_2_	Fatty acid methyl ester	33.42	1.12	33.38	2.90
12	Nonadec-1-ene	266	C_19_H_38_	Essential oil	34.69	0.24	34.7	0.50
13	9,12, octadecanoic acid, methyl ester	294	C_19_H_34_O_2_	Fatty acid methyl ester	36.85	0.10	36.73	4.12
14	3,7,11,15-tetramethyl-2-hexadeca-1-ol	296	C_10_H_40_O	Diterpene	37.10	0.53	37.08	8.10
15	Hexadecenoic acid, 2-hydroxyl-1-(hydroxy methyl) ethyl ester	330	C_19_H_38_O_4_	Fatty acid ethyl ester			43.99	1.12
16	Bis(2-ethylhexyl) phthalate	390	C_24_H_38_O_4_	Phthalate ester	44.62	0.1	44.6	2.11
17	Lanostan-3-yl-acetate	472	C_32_H_56_O_2_	Triterpenoid			51.39	1.1
18	Campesterol	400	C_28_H_48_O	Sterol			54.60	0.79
19	Stigmasterol	454	C_29_H_48_O	Sterol			55.09	0.35
20	Obtusifiol	426	C_30_H_50_O	Sterol			55.66	0.41
21	Beta-sitosterol acetate	456	C_31_H_52_O_2_	Sterol	56.00	0.10	56.05	2.69
22	Cholest-5-en-3-ol, 24-propylidene, (3 beta)	426	C_30_H_50_O	Sterol			56.37	0.40
23	Ergost-25-ene-3,5,6,12-tetrol, (3.beta.,5.apha.,6.beta.,12.beta.)	448	C_28_H_48_O	Sterol	57.6	0.25	56.74	0.76
24	9,19-Cyclolanost-24-en-3-ol, (3.beta.)	426	C_30_H_50_O	Triterpene			57.45	1.05
25	Betulinaldehyde	440	C_30_H_48_O_2_	Triterpenes			59.64	4.24
26	Longifolenaldehyde	220	C_15_H_24_O	Oxygenated sesquiterpenes			60.19	1.08
27	Thunbergol	290	C_20_H_34_O	Diterpene			60.62	0.85

**Table 3:** Phenolic compound identification of methanol extract from *Calystegia silvatica* leaves and stems via HPLC.

					Leaves		Stems	
No.	Compounds	MW	M.F.	Category	Rt	mg/100 g D. W	Rt	mg/100 g D. W
1	Gallic acid	170	C_7_H_6_O_5_	Phenolic acids	3.31	1.05	3.31	0.50
2	Chlorogenic acid	354	C_16_H_18_O_9_	Phenylacrylate polyphenol compound	4.03	1.78	4.04	0.42
3	Catechin	290	C_15_H_14_O_6_	Polyphenol	4.57	0.19	4.63	0.24
4	Methyl gallate	184	C_8_H_8_O_5_	Gallate ester	5.66	0.06	5.71	0.10
5	Caffeic acid	180	C_9_H_8_O_4_	Polyphenol	6.14	0.01	6.14	0.003
6	Pyrocatechol	110	C_6_H_6_O_2_	Phenolic compounds	6.69	3.14	6.69	0.36
7	Ellagic acid	302	C_14_H_6_O_8_	Tannins	8.23	0.69	8.46	0.21
8	Coumaric acid	164	C9H8O3	Phenolic compound	8.82	2.40	8.81	0.19
9	Vanillin	152	C_8_H_8_O_3_	Benzaldehydes	9.71	0.07	9.69	0.02
10	Ferulic acid	194	C_10_H_10_O_4_	Phenolic acid	10.22	0.28	10.21	0.06
11	Naringenin	580.5	C_27_H_32_O_14_	Flavanones	10.43	0.67	10.44	0.08
12	Quercetin	302	C_15_H_10_O_7_	Flavonoid	12.72	0.05		
	Daidzein	254	C_15_H_10_O_4_	Isoflavone			11.905	0.05
13	Cinnamic acid	148	C_9_H_8_O_2_	Monocarboxylic acid	14.01	0.01	13.51	0.001

**Table 4:** Antitumor effects of *Calystegia silvatica* methanol extract on cancer and normal cell lines.

		^a^ IC_50_ (µg/mL)	
Cell Lines	Leaves	Stems	Doxorubicin (Positive Control)
^b^ CaCo2	682 ± 55 ***	353 ± 19 ***	70.1 ± 1
^c^ HeLa	208 ± 13 ***	114 ± 5 ***	40 ± 2
^d^ PC3	336 ± 57 ***	137 ± 18 *	38 ± 2
^e^ MCF7	324 ± 17 ***	172 ± 15 ***	36 ± 6
^f^ HepG2	593 ± 22 ***	236 ± 17 ***	44 ± 3
^g^ WI38	543 ± 33 ***	408 ± 4 ***	51 ± 4

^a^ IC_50_: The half-maximal inhibitory concentration, ^b^ colon cancer (Caco2), ^c^ cervical cancer (HeLa), ^d^ prostate cancer (PC3), ^e^ breast cancer (MCF7), ^f^ hepatocellular carcinoma (HepG2) and ^g^ normal human fetal lung fibroblast (WI38). The findings are shown as mean ± standard deviation. * *p* = 0.05, ** *p* = 0.001 and *** *p* = 0.0001 show significant changes compared to a positive control. The *Calystegia silvatica* stems and leaves extracts and doxorubicin were compared using one-way analysis of variance (ANOVA), then Dunnett’s multiple comparisons test.

**Table 5:** Selectivity index values of *Calystegia silvatica* methanol extract from leaves and stems for Caco2, HeLa, PC3, MCF7 and HepG2 cancer cell lines.

	^a^ SI				
Extract	^b^ Caco2	^c^ HeLa	^d^ PC3	^e^ MCF7	^f^ HepG2
Leaves	0.8	2	1.6	1.6	0.9
Stem	1.1	3.5	3	2.3	1.7
Doxorubicin	0.7	1.2	1.3	1.4	1.1

^a^ SI: selectivity index, ^b^ colon cancer (Caco2), ^c^ cervical cancer (HeLa), ^d^ prostate cancer (PC3), ^e^ breast cancer (MCF7) and ^f^ hepatocellular carcinoma (HepG2). Compounds with values higher than 3 are more active against cancerous cells than non-cancerous (WI38) cells.

## Data Availability

Not applicable.
