# Correction: Youssef et al. Phytochemical Analysis and Profiling of Antitumor Compounds of Leaves and Stems of *Calystegia silvatica* (Kit.) Griseb. *Molecules* 2023, *28*, 630

**DOI:** 10.3390/molecules28237734

**Published:** 2023-11-23

**Authors:** Ahmed M. M. Youssef, Doaa A. M. Maaty, Yousef M. Al-Saraireh

**Affiliations:** 1 Department of Pharmacology, Faculty of Pharmacy, Mutah University, P.O. Box 7, Al-Karak 61710, Jordan; 2 Department of Botany and Microbiology, Faculty of Science, Al-Azhar University, Girls Branch, Cairo 11754, Egypt; 3 Department of Pharmacology, Faculty of Medicine, Mutah University, P.O. Box 7, Al-Karak 61710, Jordan

There were errors in the original publication [1], regarding the concentrations used in the MTT assay (Section 4.3.2) and Figure 4.

The authors wish to change the stated concentrations: 156.25, 312.5, 625, 1250, 5000 and 10,000 μg/mL. The correct concentrations are: 31.25, 62.5, 125, 250, 500 and 1000 μg/mL. A correction has been made to Section 4.3.2 (MTT Assay), paragraph number 1.

Regarding Figure 4, the authors wish to change the concentration values on the x-axes of both subfigures (A) and (B). The concentration values on the x-axes 10,000, 5000, 2500, 1250, 625 and 312.5 are corrected to 1000, 500, 250, 125, 62.5 and 31.25, and the corrected Figure 4 appears below.

The authors state that the scientific conclusions are unaffected. This correction was approved by the Academic Editor. The original publication has also been updated.

## Figures and Tables

**Figure 4:**
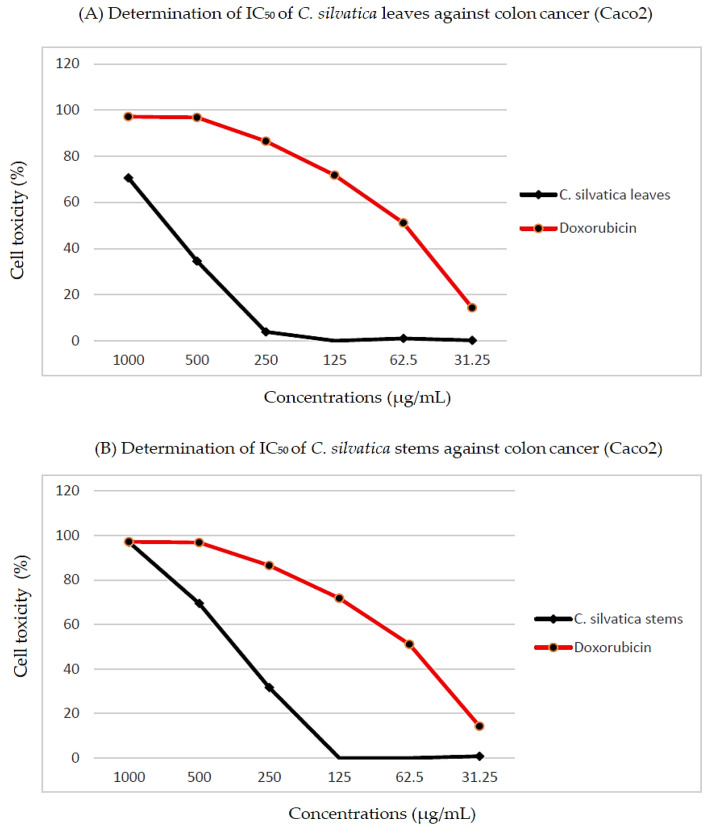
Determination of the half-maximal inhibitory concentration (IC_50_) of (**A**) *C. silvatica* leaves extract and (**B**) *C. silvatica* stems extract against colon cancer (Caco2).
